# A Cell-Based High-Throughput Screening Identified Two Compounds that Enhance PINK1-Parkin Signaling

**DOI:** 10.1016/j.isci.2020.101048

**Published:** 2020-04-11

**Authors:** Kahori Shiba-Fukushima, Tsuyoshi Inoshita, Osamu Sano, Hidehisa Iwata, Kei-ichi Ishikawa, Hideyuki Okano, Wado Akamatsu, Yuzuru Imai, Nobutaka Hattori

**Affiliations:** 1 Department of Treatment and Research in Multiple Sclerosis and Neuro-intractable Disease, Juntendo University Graduate School of Medicine, Tokyo 113-8421, Japan; 2 BioMolecular Research Laboratories, Takeda Pharmaceutical Company, Fujisawa, Kanagawa 251-8555, Japan; 3 Center for Genomic and Regenerative Medicine, Juntendo University Graduate School of Medicine, Tokyo 113-8421, Japan; 4 Department of Neurology, Juntendo University Graduate School of Medicine, 2-1-1 Hongo, Bunkyo-ku, Tokyo 113-8421, Japan; 5 Department of Physiology, Keio University School of Medicine, Tokyo 160-8582, Japan; 6 Department of Research for Parkinson's Disease, Juntendo University Graduate School of Medicine, 2-1-1 Hongo, Bunkyo-ku, Tokyo 113-8421, Japan

**Keywords:** Biological Sciences, Neuroscience, Cell Biology

## Abstract

Early-onset Parkinson's disease-associated PINK1-Parkin signaling maintains mitochondrial health. Therapeutic approaches for enhancing PINK1-Parkin signaling present a potential strategy for treating various diseases caused by mitochondrial dysfunction. We report two chemical enhancers of PINK1-Parkin signaling, identified using a robust cell-based high-throughput screening system. These small molecules, T0466 and T0467, activate Parkin mitochondrial translocation in dopaminergic neurons and myoblasts at low doses that do not induce mitochondrial accumulation of PINK1. Moreover, both compounds reduce unfolded mitochondrial protein levels, presumably through enhanced PINK1-Parkin signaling. These molecules also mitigate the locomotion defect, reduced ATP production, and disturbed mitochondrial Ca^2+^ response in the muscles along with the mitochondrial aggregation in dopaminergic neurons through reduced PINK1 activity in *Drosophila*. Our results suggested that T0466 and T0467 may hold promise as therapeutic reagents in Parkinson's disease and related disorders.

## Highlights

• A high-throughput drug discovery system for PINK1-Parkin signaling was developed
• The system identified two compounds that activate PINK1-Parkin signaling
• Two compounds activated Parkin in human dopaminergic neurons and myoblasts
• Two compounds improved mitochondrial functions of *PINK1*-knockdown *Drosophila*

## Graphical Abstract

**Figure:**
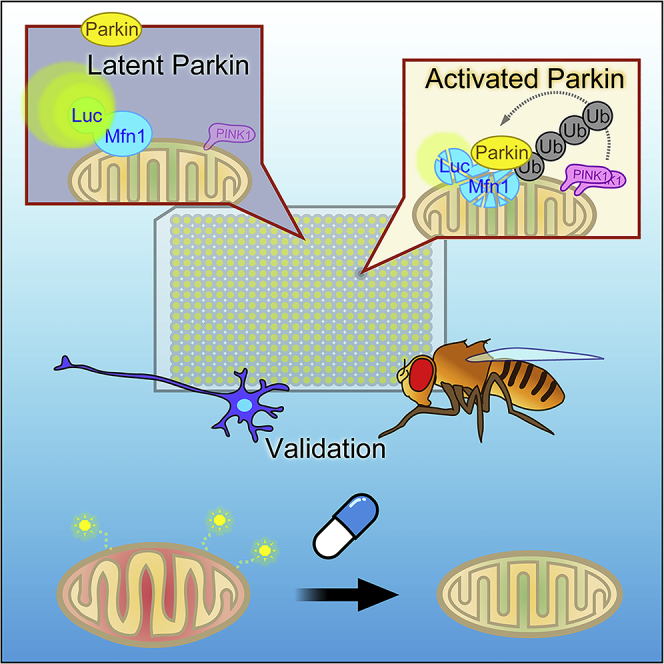

## Introduction

Homozygous or compound heterozygous mutations of genes encoding PINK1 and Parkin lead to the selective degeneration of midbrain dopaminergic neurons and cause autosomal recessive early-onset Parkinson's disease (PD) (Kitada et al., 1998, Valente et al., 2004). *Drosophila* and mammalian cell studies revealed that PINK1 and Parkin have roles in mitochondrial quality control (Clark et al., 2006, Matsuda et al., 2010, Narendra et al., 2010, Park et al., 2006, Yang et al., 2006). Subsequent studies using animal models for accelerated mitochondrial genomic error accumulation (Pickrell et al., 2015) and for mitochondrial stress through unfolded proteins (Pimenta de Castro et al., 2012) support the notion that PINK1 and Parkin maintain dopaminergic neuron survival through correcting the dysfunctional mitochondrial pool.

The PINK1 mitochondrial serine/threonine protein kinase is constitutively degraded by a combination of mitochondrial proteases and the ubiquitin-proteasome pathway in a mitochondrial membrane potential (ΔΨm)-dependent manner (Jin et al., 2010). ΔΨm reduction due to mitochondrial damage leads to PINK1 accumulation and activation at the outer mitochondrial membrane, preventing the ΔΨm-dependent import of PINK1 to the internal mitochondrial compartment (Jin et al., 2010, Okatsu et al., 2015). Activated PINK1 phosphorylates the Parkin ubiquitin ligase (E3) and Ubiquitin (Kane et al., 2014, Kondapalli et al., 2012, Koyano et al., 2014, Ordureau et al., 2014, Shiba-Fukushima et al., 2012, Shiba-Fukushima et al., 2014). Latent Parkin, in the cytosol, is activated and relocalized to the outer mitochondrial membrane, ubiquitinating mitochondrial proteins such as Mitofusin and Miro (Liu et al., 2012, Tanaka et al., 2010, Wang et al., 2011). Mitochondrial protein ubiquitination promotes mitochondrial recruitment of autophagy regulators and receptors such as TBK1 and optineurin (Heo et al., 2015, Matsumoto et al., 2015, Richter et al., 2016). The ubiquitination and subsequent degradation of Mitofusin and Miro promotes mitochondrial fragmentation and suppresses mitochondrial motility, respectively, and facilitates the autophagic removal of damaged mitochondria (Deng et al., 2008, Liu et al., 2012, Wang et al., 2011, Yang et al., 2008, Ziviani et al., 2010).

We developed a cell-based high-throughput screening (HTS) system to identify compounds (cpds) that could activate PINK1-Parkin signaling. Two unique cpds, T0466 (also known as compound 1) and T0467, were identified and characterized (Hildebrand et al., 2014). Both cpds successfully induced Parkin mitochondrial translocation in dopaminergic neurons differentiated from iPS cells (iPSCs) without obvious ΔΨm reduction and cell toxicity and eliminated unfolded mitochondrial protein caused by a truncation of ornithine carbamoyltransferase (ΔOTC) from mitochondrial pools. In the *Drosophila PINK1* model, both cpds improved the motor defects, aggregated mitochondrial morphology and decreased ATP production caused by reduced PINK1 activity. Moreover, T0467 suppressed the altered mitochondrial Ca^2+^ response caused by reduced PINK1 expression. These cpds may be promising drug candidates for diseases associated with mitochondrial damage.

## Results

### Development of a Cell-Based HTS System for PINK1-Parkin Activation Drugs

**Figure 1:**
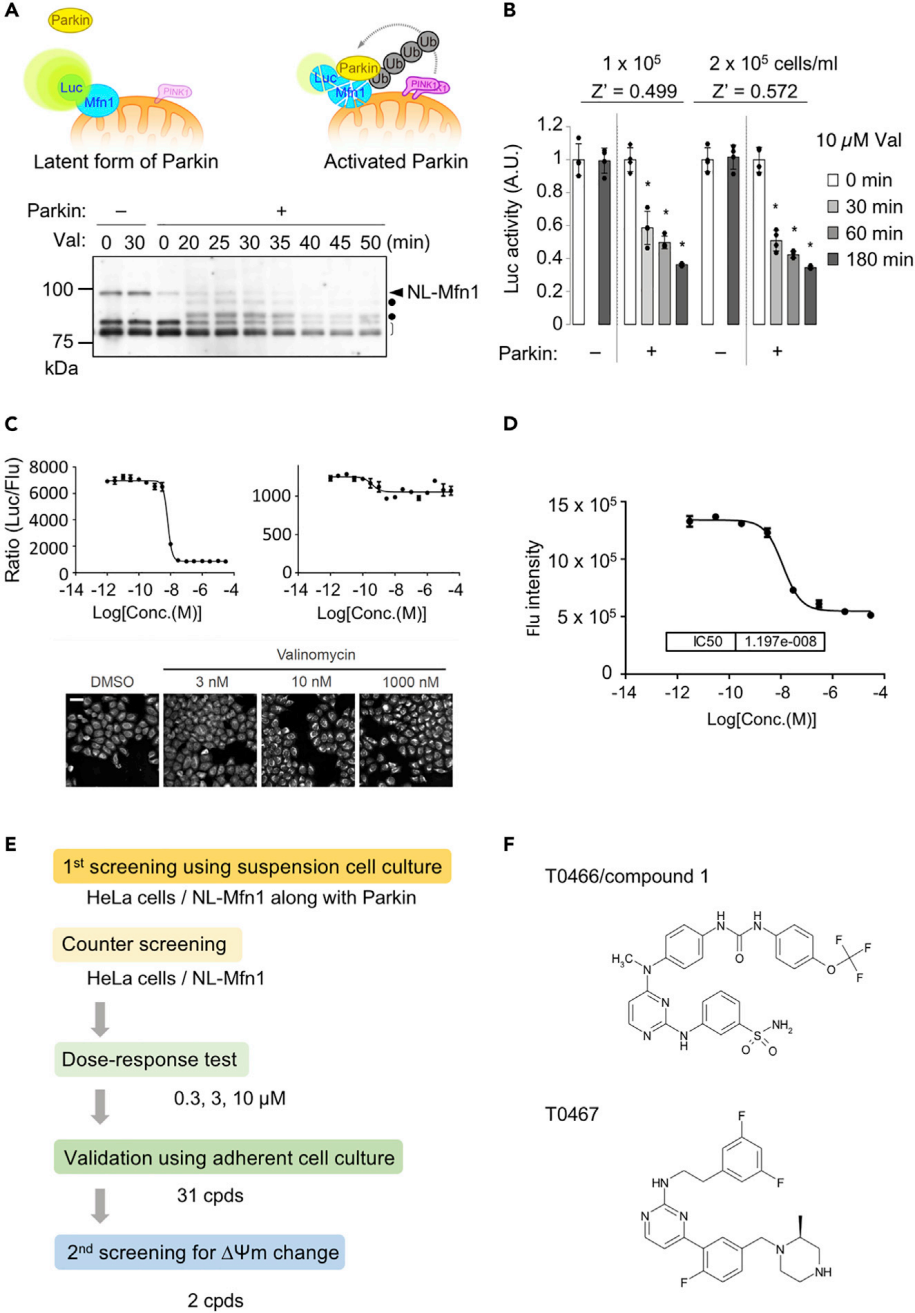
Development of the NL-Mfn1 Screening System (A) (Top) Schema of the NL-Mfn1 reporter assay to monitor Parkin activity. (Bottom) Time course analysis of NL-Mfn1 degradation after PINK1 activation by valinomycin (Val) treatment. Lysate of HeLa cells expressing NL-Mfn1 in the presence or absence of Parkin treated with 10 μM Val were analyzed by western blot with anti-Mfn1. NL-Mfn1 (arrowhead), endogenous Mfn1 (open bracket) and ubiquitinated Mfn1 (dots) are shown. (B) Validation of the NL-Mfn1 reporter assay using Val. NL activity was normalized using fluorescence signals monitoring cell viability and density in 96-well plates. The value of non-treatment (0 min) for each cell line was set as 1. Z′-factors for the given cell densities are also shown. Data are presented as mean ± SEM from four independent samples. ∗p < 0.01 by one-way ANOVA with Tukey-Kramer test. (C) Val dose response in the NL-Mfn1 reporter assay. (Top) Graphs (mean ± SD, n = 2 independent samples) representing NL activity normalized using the fluorescence signals of HeLa cells expressing NL-Mfn1 in the presence (left) or absence (right) of Parkin and treated with the indicated doses of Val. (Bottom) PINK1 accumulation in reporter cells with Parkin under the same conditions. Scale bar, 50 μm. (D) ΔΨm assay validation using MitoTracker Red. Cells were treated as in (C). The IC_50_ was 1.197 × 10^−8^ M, at which the NL-Mfn1 reporter fully detected Parkin activation (see graphs in [C]). Data are presented as mean ± SD from eight independent samples. (E) Screening flow. See Methods for details. (F) Chemical structure of T0466 and T0467. See also Figure S1.

Mitofusin family member, Mitofusin 1 (Mfn1), is rapidly degraded by active Parkin in association with mitochondrial depolarization-dependent PINK1 activation (Shiba-Fukushima et al., 2012). We developed a cell-based reporter system, described herein, that utilizes NanoLuc (England et al., 2016). Mfn1 was N-terminally fused with a newly developed luciferase, NanoLuc (NL), and this reporter was named NL-Mfn1. We generated HeLa cells stably expressing NL-Mfn1 with or without Parkin. NL-Mfn1 and endogenous Mfn1 were rapidly degraded in the presence of Parkin after mitochondrial depolarization by valinomycin (val) (Figure 1A). We isolated single cell clones expressing NL-Mfn1 to ensure robustness for large-scale drug screening. We evaluated the sensitivity of the NL-Mfn1 screening system using val, a PINK1-Parkin signaling activator. NL activity was sequentially measured to assess Mfn1 expression levels, and fluorescence signals were used to monitor cell density (see detail in Materials and Methods). The NL-Mfn1 screening system reliably detected Mfn1 degradation only in the presence of Parkin. This was observed by reduced NL activity in cells grown at two different densities, with a Z′-factor value of 0.50–0.57 (Figure 1B). The sensitivity of the NL-Mfn1 screening system was assessed using a series of val dilutions. The NL reporter responded in the presence of Parkin following treatment with over 10 nM of val. Under these conditions, PINK1 accumulation and ΔΨm reduction were observed (Figures 1C and 1D). The NL-Mfn1 system also worked using cell suspensions, the sensitivity of which was comparable with that measured using adherent cells (Figure S1).

We applied the NL-Mfn1 system to a 1,536-well plate format and performed meso-scale drug discovery using the Takeda compound library (Figure 1E). Thirty-one candidates were assessed for ΔΨm independence using the ΔΨm assay. Two cpds, T0466 and T0467, were obtained as drug candidates for PINK1-Parkin signaling activation (Figures 1E and 1F).

### T0466 and T0467 Stimulate the Mitochondrial Translocation of Parkin in a PINK1-Dependent Manner

**Figure 2:**
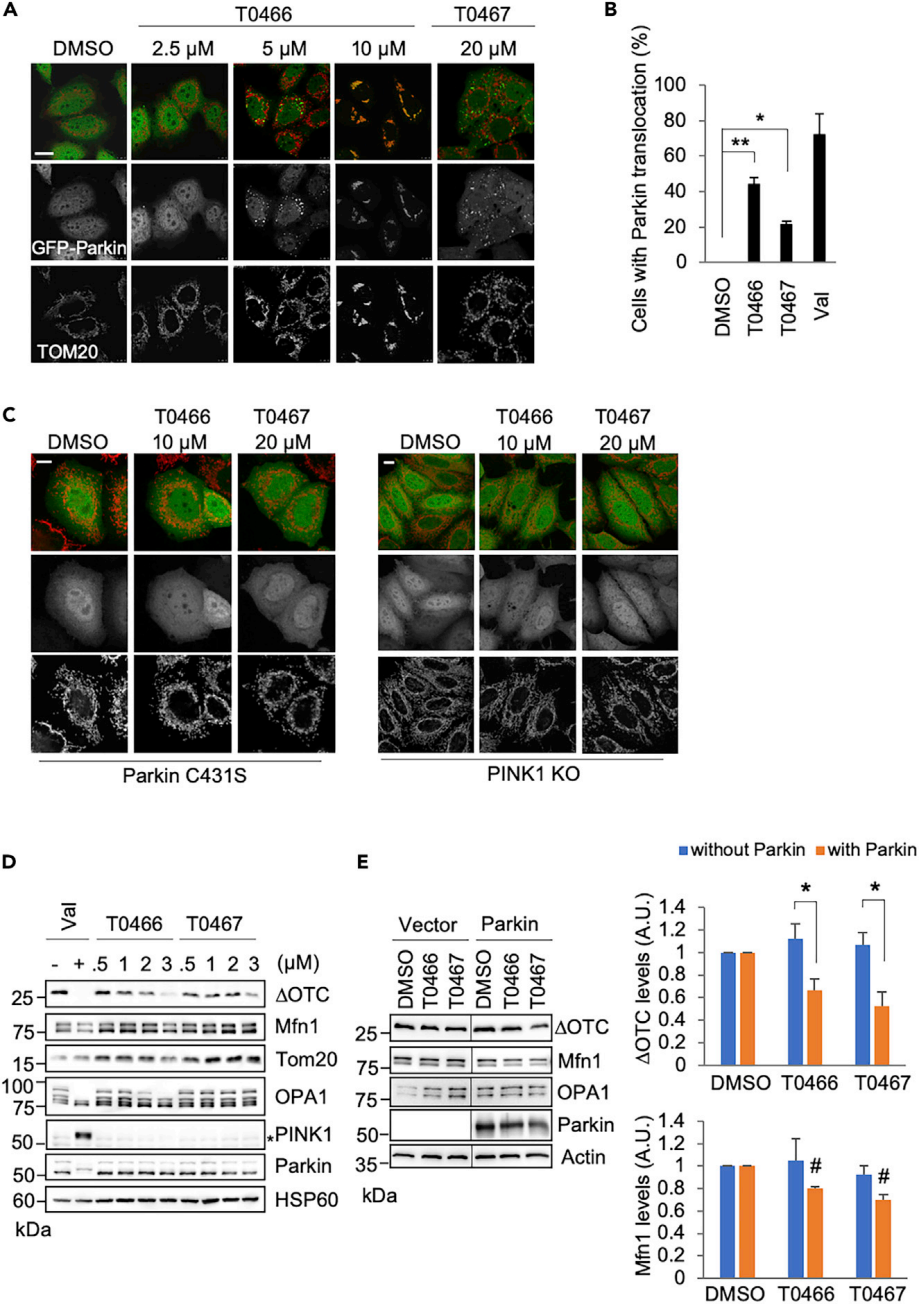
T0466 and T0467 Activate Parkin, Which Reduces an Unfolded Mitochondrial Protein (A) T0466 and T0467 stimulate mitochondrial translocation of Parkin in HeLa cells. HeLa/GFP-Parkin cells were treated with the cpds at the indicated concentrations for 3 h. DMSO treatment served as a mock control. Mitochondria were visualized with anti-TOM20 staining. Scale bar, 20 μm. (B) Mitochondrial translocation efficiency of Parkin by the cpds. HeLa/GFP-Parkin cells were treated with 5 μM T0466 and 20 μM T0467 for 3 h. The graph (mean ± SEM, n = 3 biological replicates) represents the percentage of cells with GFP-Parkin colocalized with TOM20. Cells treated with a mitochondrial uncoupling reagent Val (10 μM) served as the positive control. ∗p < 0.005, ∗∗p < 0.0001 versus DMSO using Dunnett's test. (C) Parkin mitochondrial translocation by T0466 and T0467 is Parkin E3 activity- and PINK1-dependent. HeLa cells transfected with an E3 dead form of GFP-Parkin C431S (left) and *PINK1*-deficient HeLa cells (right, PINK1 KO) were treated with T0466 or T0467 as in (A). Scale bars, 25 μm. (D) Unfolded mitochondrial protein (ΔOTC) is reduced by T0466 and T0467. ΔOTC/HeLa-TetOn cells with the stable expression of YFP-Parkin were treated with doxycycline (DOX, 1 μg/mL) for 72 h to induce mitochondrial ΔOTC expression. After removal of DOX, cells were treated with the indicated concentration of drugs for 8 h. Asterisk, non-specific. (E) T0466 and T0467 promote ΔOTC degradation in the presence of Parkin. ΔOTC/HeLa-TetOn cells transfected with a mock vector or Parkin were treated with DOX (1 μg/mL) for 24 h. After removal of DOX, cells were treated with 2 μM T0466 or 3 μM T0467 for 8 h. The relative band intensities of ΔOTC in 0.5% Triton X-100-insoluble fraction normalized with actin and of Mfn1 normalized with tubulin in 0.5% Triton X-100-soluble fraction are represented here (mean ± SEM, n = 4 biological replicates). ∗p < 0.05 by two-tailed Student's t test; #p < 0.01 versus DMSO with Parkin using Dunnett's test. See also Figures S2 and S3.

We tested whether T0466 and T0467 activate Parkin mitochondrial translocation. HeLa cells stably expressing GFP-Parkin (HeLa/GFP-Parkin cells) were treated with T0466 and T0467 at different concentrations. Over 5 μM of T0466 sufficiently stimulated the mitochondrial translocation of GFP-Parkin 3–8 h after treatment, whereas over 12 μM of T0467 was required for Parkin translocation (Figures 2A, S2A, and S2B). When HeLa/GFP-Parkin cells were treated with 5 μM T0466 or 20 μM T0467 for 3 h, GTP-Parkin was translocated to the mitochondria in approximately 44% and 21% of cells, respectively (Figure 2B). However, Parkin translocation by T0466 or T0467 did not occur when the E3-dead form of Parkin was expressed in place of wild-type Parkin, or in the absence of PINK1 activity, suggesting that PINK1 and Parkin activities are required for this effect (Figure 2C). Simultaneous treatment with a previously characterized PINK1 activation molecule, kinetin triphosphate (KTP), did not enhance T0466 or T0467 efficacy (Figure S2A) (Hertz et al., 2013).

Parkin activation relieves mitochondrial unfolded protein stress presumably through the mitophagic removal of mitochondria with accumulated unfolded proteins (Jin and Youle, 2013). ΔOTC expression results in Triton X-100 insoluble protein aggregates in the mitochondrial matrix, leading to PINK1 activation and subsequent Parkin-mediated mitophagy without ΔΨm reduction (Jin and Youle, 2013). We estimated the effects of T0466 or T0467 in this context. The mitochondrial inner membrane protein, OPA1, is processed at multiple sites in a ΔΨm reduction-dependent manner. OPA1 western blots can be used to monitor subtle ΔΨm changes (Ishihara et al., 2006). Monitoring of OPA1 processing revealed that higher T0466 concentrations reduced ΔΨm. However, a dose-dependent ΔOTC decrease was observed at T0466 concentrations as low as 1 μM, at which OPA1 was not processed in HeLa cells (Figure 2D). Moreover, T0467 induced the removal of ΔOTC without OPA1 processing, suggesting that these cpds facilitate Parkin activation independently of ΔΨm status. Consistent with these observations, these cpds did not elevate PINK1 levels, indicating that the molecular targets of these cpds are likely to be a protein other than PINK1. Using the cpds at concentrations that barely affected ΔΨm in HeLa cells (i.e., 2 μM for T0466 and 3 μM for T0467), we confirmed the Parkin dependence of ΔOTC degradation (Figure 2E). Mild degradation of Mn1 was also observed in the presence of Parkin. We next investigated the possibility that these two cpds directly activate Parkin. However, these cpds failed to stimulate Parkin E3 activity *in vitro* (data not shown), and there was no evidence that the cpds directly bind to Parkin (Figure S2C).

Previous studies have shown that T0466 has type II kinase inhibitor properties and could potentially target Ser/Thr and Tyr protein family kinases, including the MLKL pseudokinase (Hildebrand et al., 2014, Ma et al., 2016). Type II kinase inhibitors occupy the adenosine pocket of kinase domains, inducing a configuration change of the key residues required for kinase activity (Dar and Shokat, 2011). We tested the possibility that MLKL negatively regulates PINK1-Parkin signaling by monitoring the mitochondrial relocation of MLKL during PINK1-Parkin activation. GFP-MLKL was localized in the cytosol with occasional punctate signals. The subcellular localization of GFP-MLKL was not altered by T0466 or by mitochondrial uncoupling treatment (Figure S3A). Moreover, MLKL overexpression did not affect the mitophagy time course, suggesting that MLKL is unlikely to be a T0466 target in PINK1-Parkin signaling (Figure S3B).

We tested whether T0466 affects the activity of known kinases involved in PINK1-Parkin signaling, including PINK1 (Kondapalli et al., 2012, Shiba-Fukushima et al., 2012), TBK1 (Heo et al., 2015), and protein kinase A (PKA) (Akabane et al., 2016). *In vitro* kinase assays indicated that T0466 neither activated nor inhibited PINK1 kinase activity in the presence or absence of ATP (Figure S3C). T0466 treatment did not affect TBK1 activation in HeLa cells (Figure S3D). PKA negatively regulates PINK1 levels through MIC60 phosphorylation (Akabane et al., 2016). *In vitro* kinase assays showed that T0466 does not affect MIC60 phosphorylation by PKA (Figure S3E). These results suggest that T0466 does not modulate PINK1, TBK1, and PKA kinase activities.

### T0466 and T0467 Activate Parkin in Dopaminergic Neurons

**Figure 3:**
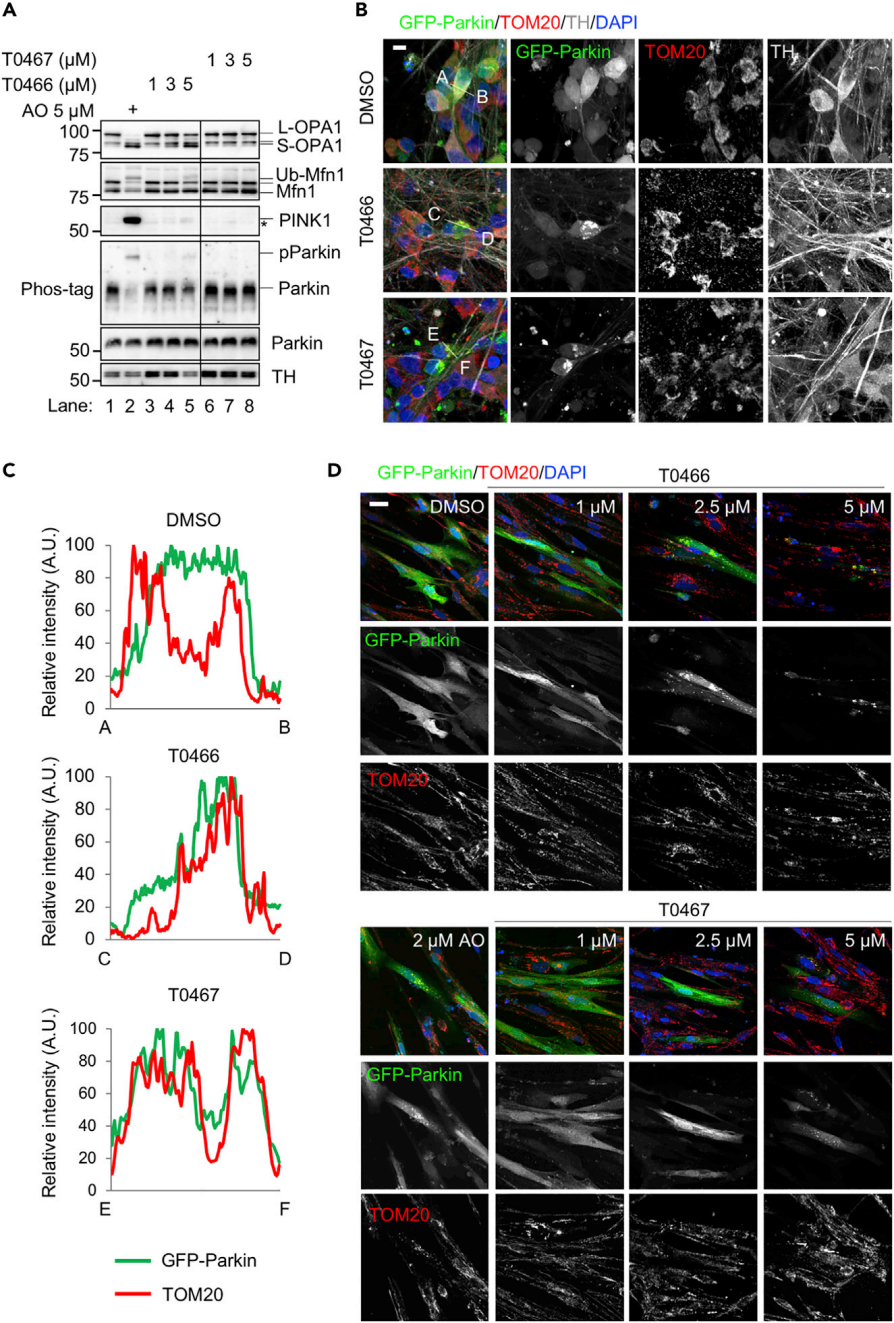
T0466 and T0467 Activate Parkin in Human Dopaminergic Neurons and Myoblasts (A) Effects of T0466 and T0467 on the ΔΨm of dopaminergic neurons. Dopaminergic neurons differentiated from human iPSCs were treated with T0466 and T0467 at the indicated concentration for 8 h. Antimycin A and oligomycin A (AO) were used as mitochondrial uncoupling reagents. Asterisk, non-specific. (B and C) T0466 and T0467 stimulate Parkin mitochondrial translocation in dopaminergic neurons. (B) Dopaminergic neurons, with virally introduced GFP-Parkin (green), were treated with 1 μM T0466 or 2.5 μM T0467 for 8 h. Single channel images for GFP-Parkin and TOM20 are also shown (grayscale). Scale bar, 10 μm. (C) Line profiles of fluorescence intensity along cross-sections in the images shown in (B). A.U., arbitrary units. (D) Myotubes forming from skeletal myoblasts, with virally introduced GFP-Parkin (green), were treated with the compounds at the indicated concentrations for 8 h. Mitochondria were visualized using anti-TOM20 staining (red). Scale bar, 25 μm. See also Figure S4.

We examined the effects of T0466 and T0467 on dopaminergic neurons, which are degenerated during the development and progression of PD. We first tested the effects of T0466 and T0467 on cell toxicity and mitochondrial functions. Treatments with T0466 and T0467 at concentrations of 0.1–1 μM did not show any cell toxicity by 48 h (Figure S4A). ATP production was moderately stimulated by lower concentrations (0.1–0.6 μM) of both cpds at 24 h, whereas treatment with 1 μM T0466 mildly reduced ATP production at 24 and 48 h (Figure S4B). We evaluated the effects of the cpds on ΔΨm in dopaminergic neuron cultures at higher concentrations. Treatment with more than 3 μM of T0466 for 8 h caused a small increase in OPA1 processing and low levels of PINK1 accumulation (Figure 3A, lanes 4 and 5), whereas 5 μM T0467 did not affect OPA1 processing and PINK1 levels (Figure 3A, lane 8). ATP production was compromised by ≥ 2.5 μM T0466 without acute cytotoxicity (Figures S4C and S4D). These results indicated that T0466 and T0467, at ≤1 μM and ≤2.5 μM, respectively, do not appear to be associated with occasional PINK1 activation by ΔΨm reduction and are appropriate for dopaminergic neuron cultures.

Parkin activation was monitored by its mitochondrial translocation. GFP-Parkin relocation from the whole cytosol to the punctate structures in dopaminergic neuron cultures was observed after treatment with 1 μM T0466 or 2.5 μM T0467 for 8 h, and the Parkin foci were overlapped with TOM20 signals, suggesting that Parkin activated by the drug treatment was translocated to the mitochondria (Figures 3B and 3C).

Parkin-mediated mitophagy is proposed to be involved in cardiomyocyte development and prevents aging-related loss of muscle mass and strength (Chen and Dorn, 2013, Leduc-Gaudet et al., 2019). Mitochondrial translocation of GFP-Parkin was also observed in myoblasts at higher concentrations (>2.5 μM and >5 μM for T0466 and T0467, respectively) than those required in dopaminergic neurons (Figure 3D).

### T0466 and T0467 Mitigate Phenotypes Caused by Reduced PINK1 Activity in *Drosophila*

**Figure 4:**
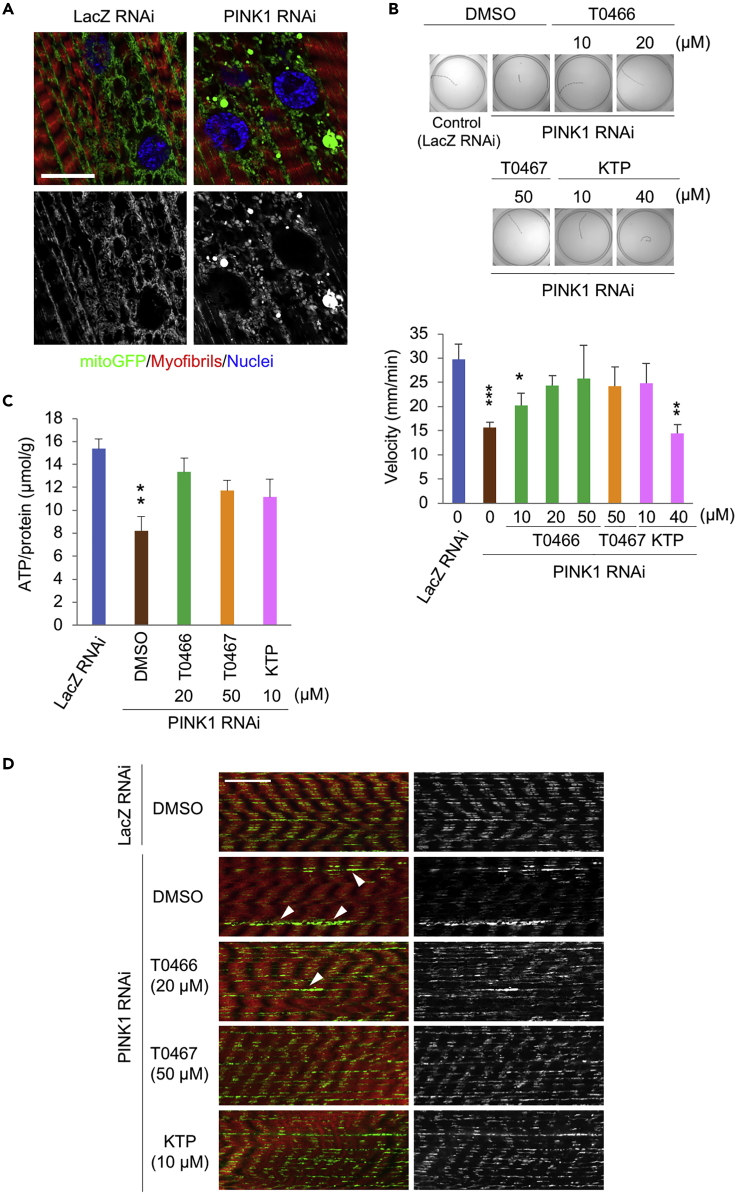
T0466 and T0467 Improve the Locomotion Defects Caused by the Reduced PINK1 Activity in *Drosophila* (A) Mitochondrial morphological changes caused by PINK1 inactivation in the larval body-wall muscles. Mitochondria were visualized by mitoGFP (green). Myofibrils and nuclei were stained with TRITC-phalloidin (red) and DAPI (blue), respectively. Scale bar, 20 μm. (B) Reduced PINK1 activity-mediated locomotion deficiency is mitigated by T0466 and T0467. Third-instar larvae treated with DMSO or the indicated drugs were placed in the center of 100-mm-diameter dishes and their movement recorded over 2 min using a CCD camera (images). The velocity over the last minute was graphed (mean ± SEM, n = 6–27 flies each). ∗p < 0.05, ∗∗p < 0.01, ∗∗∗p < 0.0001 versus LacZ RNAi by Dunnett's test. (C) ATP production in PINK1 RNAi flies is improved by T0466 and T0467 as well as KTP. ATP concentration was determined in the whole bodies of the third-instar larvae, normalizing with that of tissue soluble proteins. ∗∗p < 0.001 versus LacZ RNAi by Dunnett's test (mean ± SEM, n = 8 flies each). (D) Mitochondrial aggregation in larval body-wall muscles caused by PINK1 inactivation was improved after the administration of T0466, T0467, or KTP. Mitochondria were visualized by mitoGFP (green) and counterstained with TRITC-phalloidin (red). Single channel images of mitoGFP are also shown (grayscale). Typical mitochondrial phenotypes observed in *PINK1 RNAi* flies are reduced mitoGFP signals and aggregated mitochondria (arrowheads). Scale bar, 20 μm. Genotypes used here are *UAS-LacZ RNAi/+; MHC-GAL4/UAS-mitoGFP* (LacZ RNAi) and *MHC-GAL4, UAS-PINK1 RNAi/UAS-mitoGFP* (PINK1 RNAi). See also Figures S5A and S5B.

We assessed the effects of the two cpds on the PINK1-Parkin mitochondrial quality control pathway *in vivo*. Unlike rodent *PINK1* or *Parkin* models, *Drosophila* models exhibit obvious mitochondrial degeneration (Clark et al., 2006, Park et al., 2006, Yang et al., 2006). Flies do not consume any food or water for 3.5–4.5 days during pupation, making the evaluation of drug efficacy in adult flies just after eclosion difficult. We evaluated the efficacy of T0466, T0467, and KTP using larva, owing to their constant feeding behavior. We employed muscle-specific *PINK1* knockdown flies, which showed mitochondrial degeneration (Figure 4A and Videos S1 and S2) and locomotion defects at the third instar larval stage (Figure 4B). Inactivation of *PINK1* in the larval muscles affected crawling activity and reduced the velocity of locomotion to approximately 50% of that of control *LacZ* knockdown flies (Figure 4B). T0466 and T0467 significantly improved the locomotion defects in *PINK1* knockdown larvae (Figure 4B). KTP also mitigated the locomotion defects of *PINK1* knockdown larvae, but a higher dose of KTP did not (Figure 4B). ATP production in *PINK1* knockdown larvae was approximately 50% of that of *LacZ* knockdown flies and improved following T0466, T0467, and KTP administration (Figure 4C). In this context, both T0466 and T0467 did not affect the knockdown efficiency of PINK1 transcripts (Figure S5A), whereas these two cpds had a null effect on *PINK1*^*−/−*^ flies (Figure S5B), strongly suggesting that the cpds mitigate mitochondrial dysfunction through the modulation of PINK1-Parkin signaling.

Video S1. Mitochondrial Morphology of the Larval Body-Wall Muscles Expressing LacZ RNAi, Related to Figures 4A and 4D Mitochondria (green), the nuclei of muscular cells (blue), and myofibers (red) were visualized with mitoGFP, DAPI and TRITC-phalloidin, respectively. The focal plane moves from the inside to the outside of the body wall. Scale bar, 20 μm.

Video S2. Mitochondrial Morphology of the Larval Body-Wall Muscles Expressing PINK1 RNAi, Related to Figures 4A and 4D The focal plane moves from the inside to the outside of the body wall as in Video S1. Scale bar, 20 μm.

**Figure 5:**
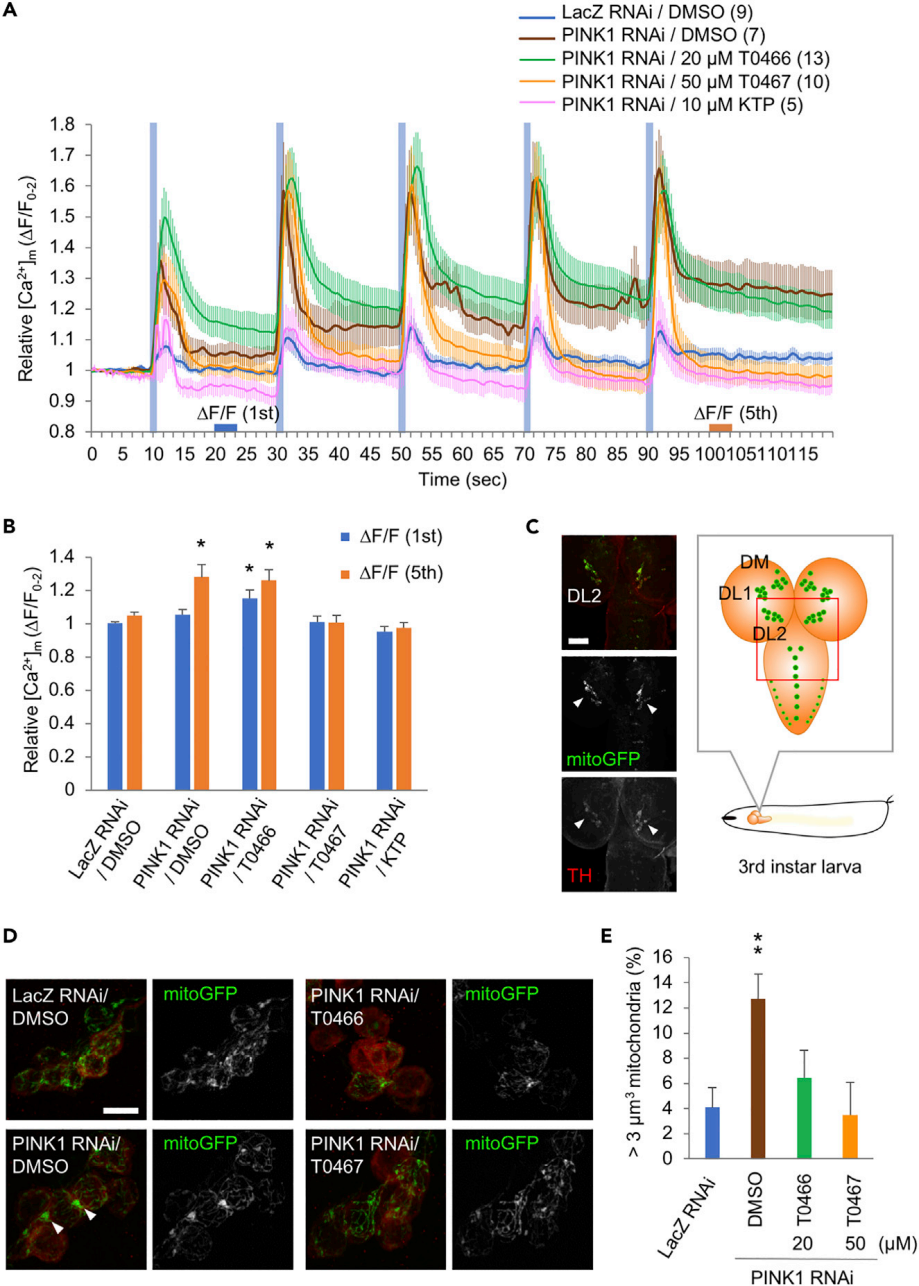
T0466 and T0467 Suppress the Mitochondrial Aggregation of Dopaminergic Neurons Caused by the Reduced PINK1 Activity in *Drosophila* (A and B) Reduced PINK1 activity-induced delay in mitochondrial Ca^2+^ efflux is improved by T0467 and KTP. (A) Traces of relative fluorescence intensity changes before and after stimulations were graphed (n = 5–13 flies). Average fluorescence intensity showing mitochondrial Ca^2+^ concentration ([Ca^2+^]_m_) from 0 to 2 s was set to 1. Blue bar indicates electrical stimulation (500 msec at 2.5 V). The number of samples analyzed are indicated in the graph legends. (B) Mitochondrial Ca^2+^ decay after the first and fifth electrical stimulation (20–22 and 100–102 s) in (A). ∗p < 0.05 versus LacZ RNAi in each time window by Dunnett's test. Data are presented as mean ± SEM in (A) and (B). (C) The DL2 clusters of dopaminergic neurons in the third-instar larval brain. (Left) The posterior brain of larva expressing mitoGFP (green) under the control of *TH-GAL4* driver. The DL2 dopaminergic neurons (arrowheads) were also visualized with anti-TH (red). Scale bar, 50 μm. (Right) The position of the larval DL2 neuron clusters is depicted. The left images correspond to the red box region. DL, dorsolateral neurons; DM, dorsomedial neurons. (D and E) Mitochondrial aggregates due to PINK1 inactivation are suppressed by T0466 and T0467. (D) The mitochondrial morphology (visualized by mitoGFP, green) of the DL2 cluster dopaminergic neurons (marked by anti-TH, red) treated with DMSO or the indicated drugs were imaged. Presented images were reconstructed from a series of z-stacked images (10–20 μm in depth). Scale bar, 10 μm. (E) Mitochondrial aggregates over 3 μm^3^ (as shown by arrowheads in [D]) were graphed (n = 11–21 flies each). ∗∗p < 0.01 versus LacZ RNAi, DMSO using Dunnett's test. All transgenes were expressed by the *MHC-GAL4* (A, B) and *TH-GAL4* drivers (C–E). See also Figure S5C.

We examined the effects of drug administration on mitochondrial morphology and function in muscles. In normal control flies (LacZ RNAi, DMSO), the mitochondria of body-wall muscles were aligned alongside the myofibrils. However, in *PINK1* knockdown flies, aggregated or irregularly aligned mitochondria were observed (Figure 4D). Mitochondrial aggregation of body-wall muscles by PINK1 inactivation was partially ameliorated by T0466 treatment and markedly improved by T0467 and KTP (Figure 4D). A maintained proton gradient across the mitochondrial inner membrane is required for ATP-dependent Ca^2+^ influx and efflux (Xing and Wu, 2018). We performed mitochondrial Ca^2+^ imaging analysis using muscular mitochondria-targeted GCaMP in larval neuromuscular junctions. Sequential electrostimulation of motor neuron nerves in muscle-specific *PINK1* knockdown flies caused a gradual increase in GCaMP intensity baseline during five consecutive stimulations, indicating a delay in mitochondrial Ca^2+^ efflux due to mitochondrial dysfunction (Figures 5A and S5C). Treatment with T0467 and KTP at concentrations most effectual in motor behavior analyses significantly improved the delay in mitochondrial Ca^2+^ decay after the stimulation-mediated Ca^2+^ spike, suggesting that T0467 and KTP alleviate PINK1 inactivation-induced mitochondrial dysfunction (Figures 5A and 5B).

We next examined whether these cpds exert a beneficial effect in dopaminergic neurons *in vivo*. Although *Drosophila* has an evolutionarily conserved blood-brain barrier (BBB), treatment with both cpds suppressed mitochondrial aggregates of larval dopaminergic neurons caused by PINK1 inactivation, suggesting that these drugs go through at least the *Drosophila* BBB (Figures 5C–5E) (Davis et al., 2019, Hindle et al., 2017, Limmer et al., 2014, Mayer et al., 2009, Zhang et al., 2018).

## Discussion

Here, we describe a robust cell-based HTS for PINK1-Parkin signaling. Using this approach, we isolated two candidate cpds. Previous studies have reported two main kinds of drugs to activate PINK1, KTP (Hertz et al., 2013) and its derivative (Osgerby et al., 2017) and niclosamide and its analogues (Barini et al., 2018). KTP is an ATP analogue that might affect a wide range of kinases and specific types of mRNA splicing (Lee et al., 2009, Wei et al., 2018). The niclosamide anthelmintic drug decreased the ΔΨm, leading to PINK1 accumulation. In contrast, T0466 and T0467 do not appear to act directly on PINK1 and have unique potential for application as therapeutic reagents in diseases associated with mitochondrial degeneration.

OPA1 processing status revealed that T0466 affected ΔΨm at concentrations >1 μM in dopaminergic neurons and HeLa cells. However, PINK1 levels at T0466 concentrations ≥1 μM were comparable with those of healthy controls. More uniquely, T0467 successfully activated mitochondrial translocation in dopaminergic neurons and myoblasts derived from iPSCs without obvious ΔΨm reduction at lower concentrations than in HeLa/GFP-Parkin cells. Moreover, these two cpds reduced the levels of the mitochondrial unfolded protein ΔOTC without ΔΨm reduction-induced PINK1 accumulation.

T0466 and T0467 did not show obvious toxicity in *Drosophila* at concentrations <50 μM. All cpds examined mitigated the PINK1 inactivation-mediated larval locomotion defects and mitochondrial morphological defects and reduced ATP production. Additionally, T0467 and KTP improved the mitochondrial Ca^2+^ response in *Drosophila* larval muscles. A recent study reported that KTP did not show beneficial effects in *PINK1* mutant mouse models, although appropriate evaluation was difficult owing to lack of PD-like phenotypes (Orr et al., 2017). Given the limitations of drug evaluation using rodent genetic PD models, our approach using a combination of dopaminergic neuron cultures from iPS cells and *Drosophila* genetic PD models could provide a new standard method to rapidly assess drug efficacy.

Dysfunction of Parkin-mediated mitochondrial maintenance is involved in the pathophysiological basis of a variety of diseases including PD, amyotrophic lateral sclerosis, diabetes, cardiomyopathy, and muscular atrophy (Billia et al., 2011, Hoshino et al., 2014, Kang et al., 2018, Kitada et al., 1998, Leduc-Gaudet et al., 2019, Palomo et al., 2018). Heterozygous mutations in PINK1 and Parkin are a reported risk for sporadic PD (Klein et al., 2007, Shulskaya et al., 2017). Previously, we reported that PINK1-Parkin activity is higher in dopaminergic neurons than in other cells differentiated from the same iPSC lines (Shiba-Fukushima et al., 2017). Thus, the cpds identified in this study might be especially effective in dopaminergic neurons affected in PD. Further studies should elucidate the molecular mechanism underlying the activation of the PINK1-Parkin pathway by these cpds. Moreover, drug optimization, including structural optimization to reduce toxicity and enhance delivery to the central nervous system, is expected to accelerate the clinical development of drugs to treat PD and related diseases.

### Limitations of the Study

Herein, we developed a cell-based screening system for cpds that activate PINK1-Parkin signaling and identified two cpds. Although these cpds are effective in mammalian cells and *Drosophila* in the presence of PINK1, this study did not determine the molecular targets of these cpds. Thus, the establishment of non-human primate models of PINK1-Parkin-associated PD that reproduce PD-like phenotypes and the evaluation of drug properties including pharmacokinetic profiles and potential adverse effects using these mammalian models are required in the future studies.

## Methods

All methods can be found in the accompanying Transparent Methods supplemental file.

## Data and Code Availability

The data that support the findings of this study are available from the corresponding author upon reasonable request.

## Supplemental Information

Document S1. Transparent Methods and Figures S1–S5
