# Supplementary material for: A Cell-Based High-Throughput Screening Identified Two Compounds that Enhance PINK1-Parkin Signaling
Source: iScience. 2020 Apr 11;23(5):101048. doi: 10.1016/j.isci.2020.101048 (PMC7183160; doi:10.1016/j.isci.2020.101048)
Supplement: Document S1. Transparent Methods and Figures S1–S5 [file mmc1.pdf]

iScience, Volume *23*

Supplemental Information

A Cell-Based High-Throughput Screening Identified Two Compounds that Enhance PINK1-Parkin Signaling

Kahori Shiba-Fukushima, Tsuyoshi Inoshita, Osamu Sano, Hidehisa Iwata, Kei-ichi Ishikawa, Hideyuki Okano, Wado Akamatsu, Yuzuru Imai, and Nobutaka Hattori

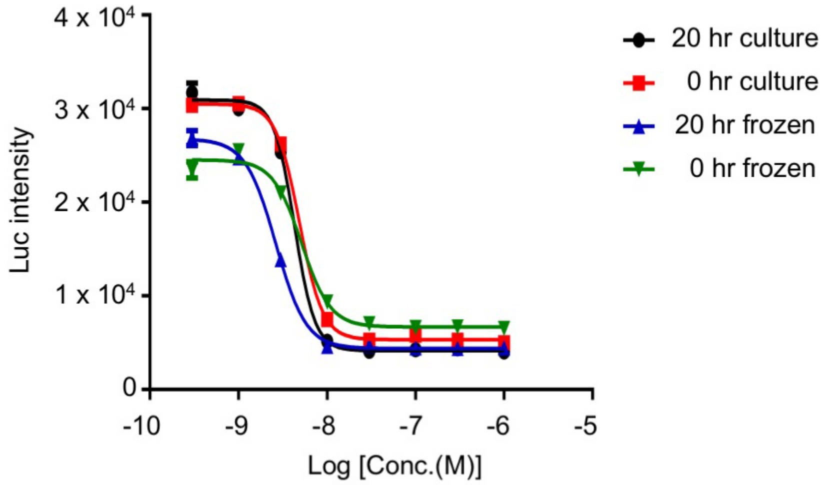

Figure S1. Validation of HTS method using suspension culture, Related to Figure 1.
The NL-Mfn1 reporter assay works using cell suspensions. Reporter cells were treated with valinomycin (Val) under four different culture conditions. Reporter assays were performed 3 h after Val addition. For the 20 h culture, growing cells on dishes were plated and cultured for 20 h before the addition of Val. For the 0 h culture, growing cells on dishes were plated and immediately treated with Val. For the 20 h frozen treatment, frozen cells were plated and cultured for 20 h before the addition of Val. For the 0 h frozen treatment, frozen cells were plated and immediately treated with Val. Data are presented as mean ± SD from 2 independent samples.

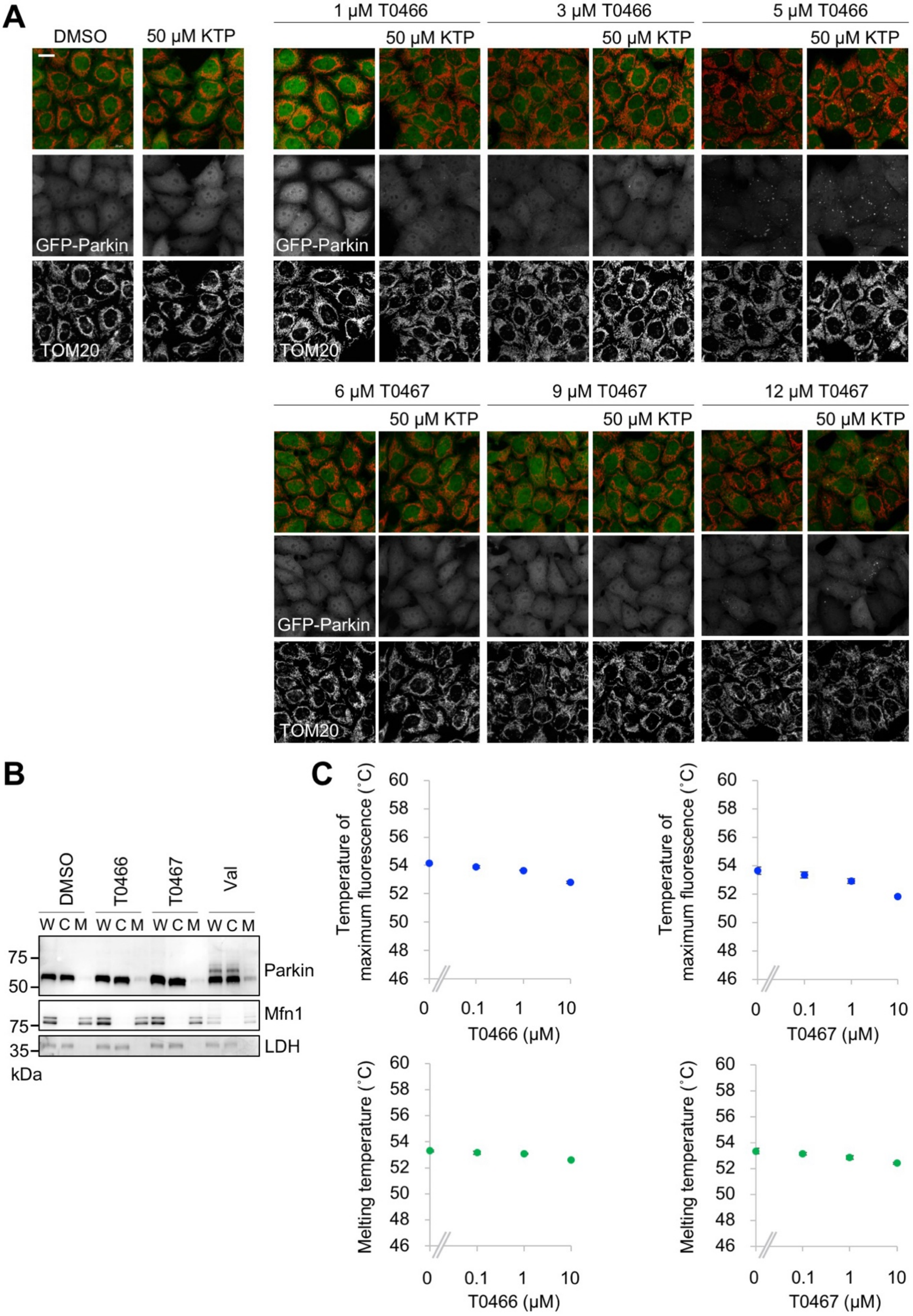
A
DMSO
50 μM KTP
1 μM T0466
3 μM T0466
5 μM T0466
GFP-Parkin
TOM20
6 μM T0467
9 μM T0467
12 μM T0467
B
DMSO
T0466
T0467
Val
W C M
Parkin
Mfn1
LDH
75
50
35
kDa
C
Temperature of maximum fluorescence (˚C)
Melting temperature (˚C)
60
58
56
54
52
50
48
46
0
0.1
1
10
T0466 (μM)
T0467 (μM)

Figure S2. Titration of T0466 and T0467 with or without KTP, Related to Figure 2.
(**A**) KTP does not potentiate the effects of T0466 and T0467 on Parkin activation. HeLa/GFP-Parkin cells were treated with T0466 and T0467 with or without 50 μM KTP at the indicated concentrations for 8 h. Treatment with 5 μM T0466 or 12 μM T0467 activated Parkin, detected as punctate GFP signals, while KTP alone did not. Mitochondria were visualized with anti-TOM20 staining. Scale bar = 20 μm.
(**B**) Biochemical fractionation of mitochondrial Parkin after drug treatment. HeLa/GFP-Parkin cells were treated with DMSO, 5 μM T0466, 20 μM T0467, or 20 μM Val for 3 h. W, whole lysate; C, cytosolic fraction; M, mitochondrial fraction. Mfn1 and Lactate dehydrogenase (LDH) served as mitochondria and cytosol markers, respectively.
(**C**) No evidence that Parkin is a target of cpds was seen. Upper graphs (mean ± SEM, n = 4 technical replicates) indicate the temperature of maximum fluorescence of SYPRO Orange at given concentrations of cpds in the thermal shift assay of Parkin. Lower graphs (mean ± SEM, n = 4 technical replicates) indicate melting temperature obtained by the first derivative of the fluorescence intensity curves. Here, 0 μM means solvent DMSO alone. The addition of cpds to Parkin did not result in a prominent shift in the temperature of the melt peak, suggesting that cpds do not bind to Parkin.

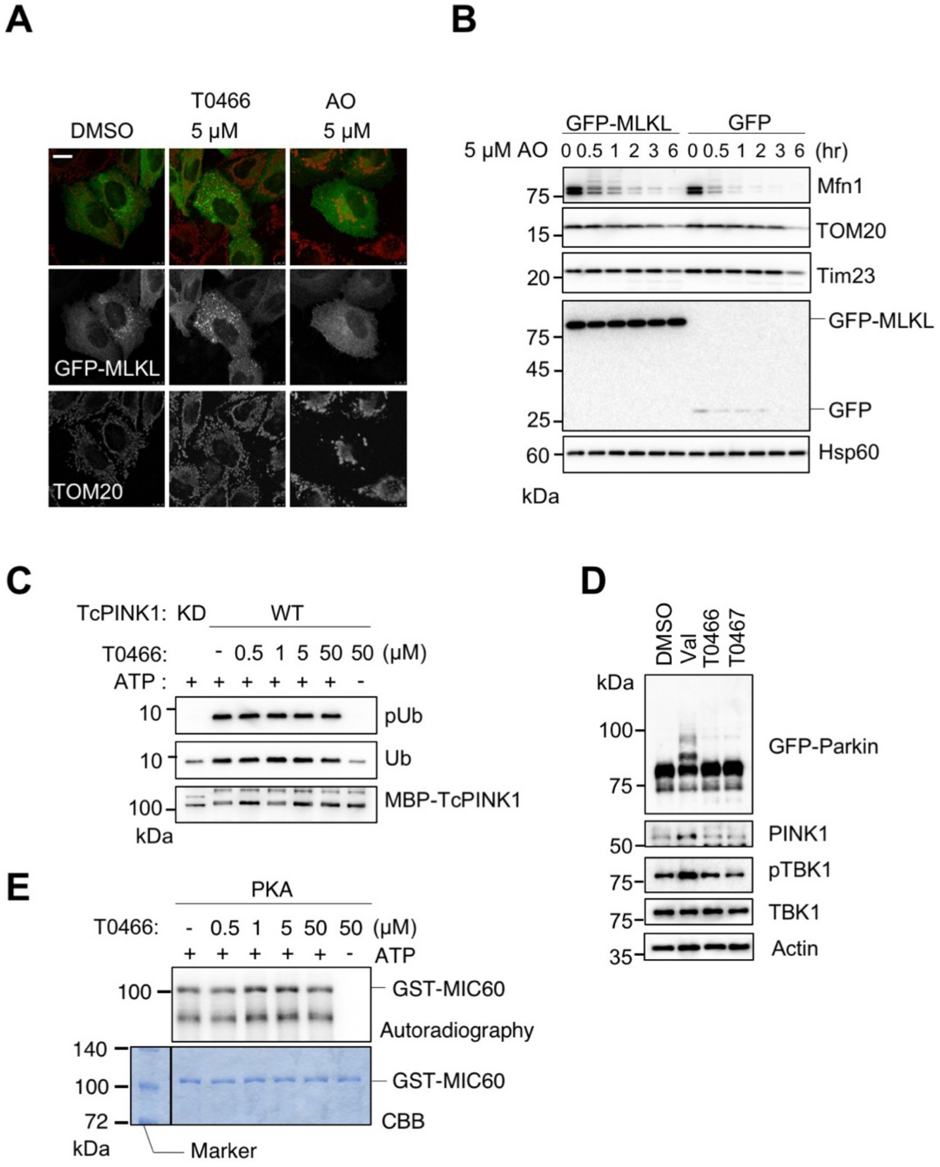

Figure S3. PINK1, TBK1, and PKA are not T0466 or T0467 targets, Related to Figure 2.
(**A**) T0466 does not alter MLKL subcellular localization. HeLa cells transfected with GFP-MLKL were treated with T0466 (5 μM) or AO (5 μM) for 3 h. Scale bar = 20 μm.
(**B**) MLKL does not affect Parkin-mediated mitophagy. Cells expressing GFP-MLKL or GFP were treated with 5 μM AO for the indicated times.
(**C**) T0466 does not modulate the kinase activity of PINK1. Recombinant TcPINK1 was preincubated with or without T0466 for 30 min at RT and further incubated with Ubiquitin (Ub) at 30˚C for 90 min in the presence or absence of 2 mM ATP. Phosphorylation of Ub at Ser65 (pUb) was detected with anti-phospho-Ser65 Ub antibody.
(**D**) T0466 and T0467 do not stimulate PINK1 accumulation and TBK1 phosphorylation. HeLa/GFP-Parkin cells were treated with 5 μM T0466 and T0467 for 3 h. The indicated proteins were analyzed by western blot.
(**E**) T0466 does not modulate PKA kinase activity. PKA was preincubated with or without T0466 for 30 min at RT and further incubated with GST-MIC60 at 30˚C for 90 min in the presence or absence of γ-^{32}P ATP. Phosphorylation of GST-MIC60 was detected by autoradiography. The amounts of GST-MIC60 were detected by Coomassie brilliant blue (CBB) staining.

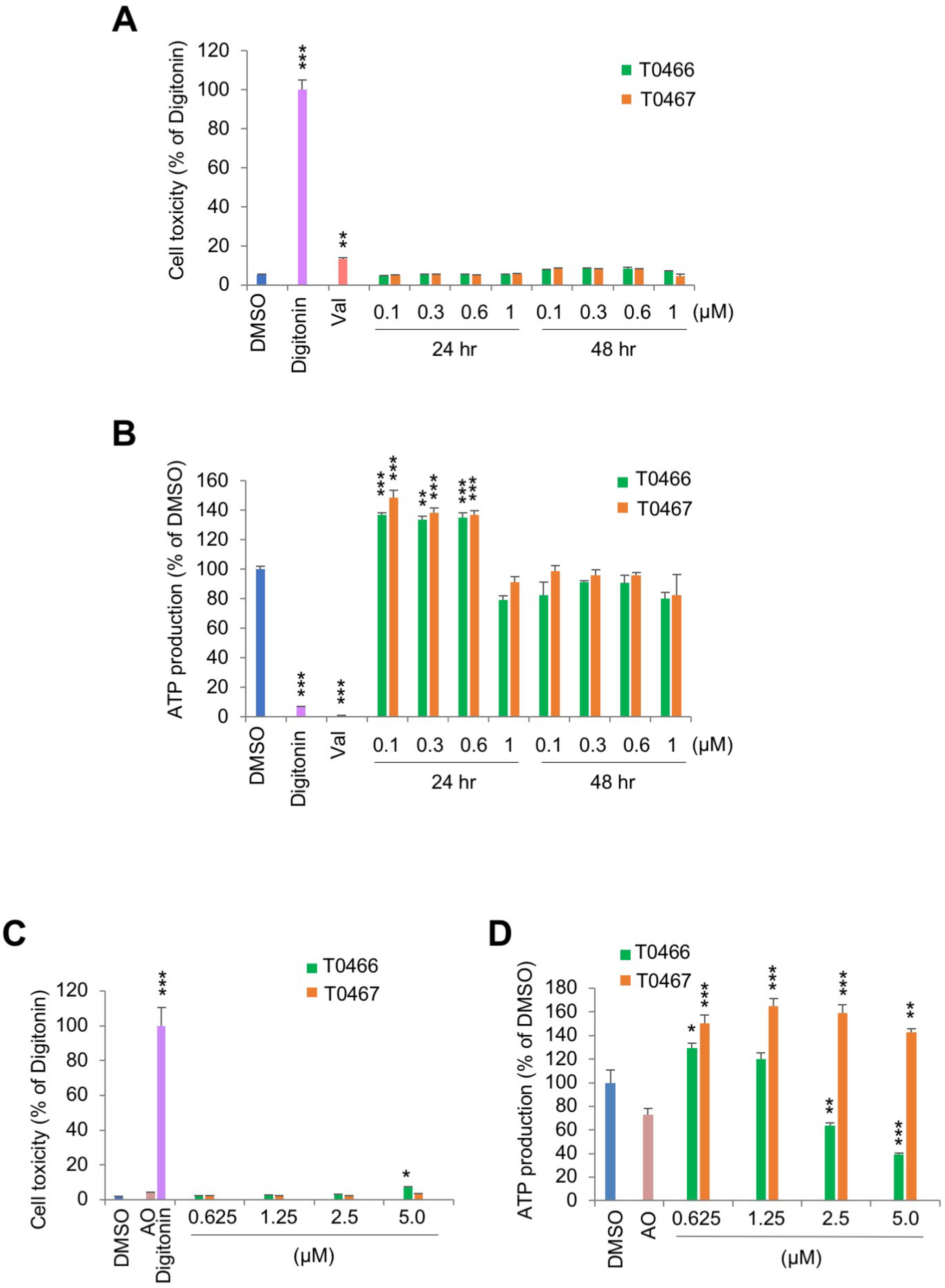

Figure S4. Effects of T0466 and T0467 on ATP production and cell toxicity in dopaminergic neuron culture, Related to Figure 3.

(**A**) Cell toxicity test of chemicals in dopaminergic neuron culture treated with chemicals at concentrations of 0.1−1 μM for the indicated times (mean ± SEM, n = 4 independent samples). DMSO and digitonin treatments served as mock and positive controls, respectively. Valinomycin (Val, 2 μM) was also put as a mitochondrial toxin treatment. **$p < 0.005$, ***$p < 0.0001$ *vs*. DMSO by one-way ANOVA with Tukey-Kramer test.

(**B**) ATP measurement in iPSC-derived dopaminergic neuron culture treated with chemicals at concentrations of 0.1-1 μM for the indicated times (mean ± SEM, n = 4 independent samples). Valinomycin (Val, 2 μM) treatment served a mitochondrial toxin control. ***$p < 0.01$, ****$p < 0.001$ *vs*. DMSO by one-way ANOVA with Tukey-Kramer test.
(**C**) Cell toxicity test of chemicals in dopaminergic neuron culture treated with chemicals at concentrations of 0.625-5 μM for 8 h (mean ± SEM, n = 4-6 independent samples). DMSO and digitonin treatments served as mock and positive controls, respectively. AO (2 μM each) treatment served a mitochondrial toxin control. *$p < 0.05$, ***$p < 0.0001$ *vs*. DMSO by one-way ANOVA with Tukey-Kramer test.
(**D**) ATP measurement in iPSC-derived dopaminergic neuron culture treated with chemicals at concentrations of 0.625-5 μM for 8 h (mean ± SEM, n = 4-6 independent samples). *$p < 0.05$, **$p < 0.01$, ***$p < 0.0001$ *vs*. DMSO by one-way ANOVA with Tukey-Kramer test.

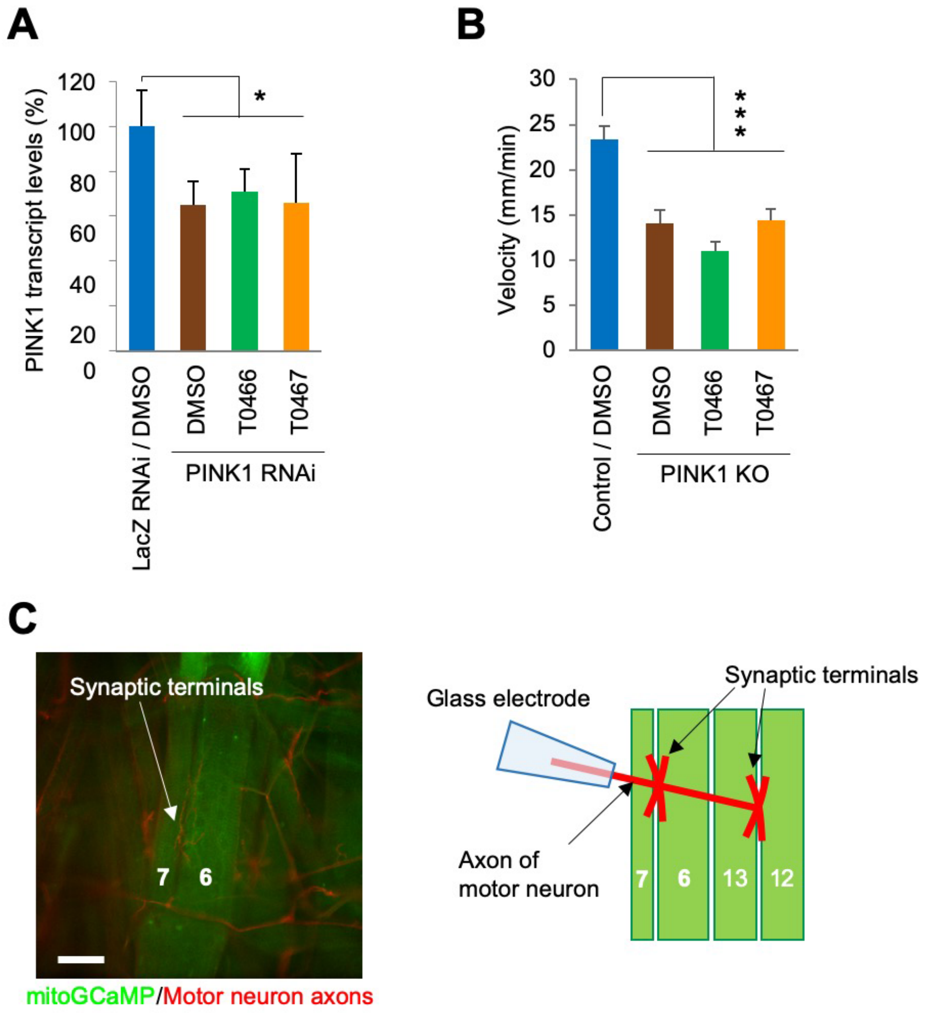

Figure S5. Mitochondrial defects in the muscles of PINK1 knockdown larvae, Related to Figures 4 and 5.

(**A**) T0466 and T0467 do not affect PINK1 knockdown efficiency. *PINK1* transcript levels normalized with housekeeping *RP49* levels in the larval body-wall muscles are represented (mean ± SEM, n = 5 biological replicates). *$p < 0.05$ using Dunnett's test.

(**B**) T0466 and T0467 do not improve the locomotion of PINK1 KO flies. The locomotion of PINK1 KO third-instar larvae treated with DMSO or the indicated drugs were recorded as in Fig. 4B and graphed (mean ± SEM, n = 11–14 flies each). ***$p < 0.0001$ using one-way ANOVA with Tukey-Kramer test.

(**C**) Larval body-wall muscles six and seven at the abdominal segment three and motor neuron axons projected to these muscles are shown. Motor neuron axons were visualized with Alexa594 conjugated anti-HRP (red). Bar = 100 µm. (right) Diagram of the mitochondrial Ca^{2+} imaging experiment. Motor neuron axons projecting into abdominal segment three muscles are stimulated by a glass suction electrode with a stimulation for 500 msec 5 times at 20 sec intervals.

Genotypes are: (**A, C**) *UAS-LacZ RNAi/+; MHC-GAL4/UAS-mitoGFP* (LacZ RNAi) and *MHC-GAL4, UAS-PINK1 RNAi/UAS-mitoGFP* (PINK1 RNAi), (**B**) *+/Y; +/+; MHC-GAL4, UAS-mitoGFP/+* (Control), *PINK1^{B9}/Y; +/+; MHC-GAL4, UAS-mitoGFP/+* (PINK1 KO). Males were discriminated by their testes.

Transparent Methods

Plasmids and cell lines

pNLF1-C (Promega) and pMXs-puro/-neo (Cell Biolabs) vectors were purchased. Complementary DNA of Human Mfn1 amplified from HeLa cells was cloned into the XhoI and NotI sites of the pCAGGS-CFP vector (a gift of Dr. Y. Ohba at Hokkaido University). The NL coding sequence was retrieved from pNLF1-C as an EcoRI/XhoI fragment. NL and Mfn1 fragments were then cloned into the EcoRI and NotI sites of the pMXs-puro vector. Human Parkin cDNA was cloned into the EcoRI and XbaI sites of pMXs-neo vector. HeLa cells were transfected with pMXs-puro-NL-Mfn1 and pMXs-neo-Parkin or pMXs-neo empty vector along with pcDNA3-Hyg-mSlc7a1-VSVG. Stable cell lines were selected with 1 μg/ml puromycin and 1 mg/ml neomycin (Shiba-Fukushima et al., 2012). Single clones stably expressing the NL-Mfn1 reporter, with or without Parkin, were then isolated and characterized. HeLa cell/NL-Mfn1/Parkin and HeLa cell/NL-Mfn1/Vector were used for HTS and counter screening, respectively. MLKL cDNA amplified from HeLa cells was cloned into the XhoI and BamHI sites of pEGFP-C1 vector. GFP-Parkin was inserted into the lentivirus vector pLVSIN-CMV-Pur for neuronal transfection (Takara Bio, Japan). HeLa cells stably expressing GFP-Parkin (HeLa/GFP-Parkin cells) were generated by retroviral infection of pMXs-puro-GFP-Parkin WT along with pcDNA3-Hyg-mSlc7a1-VSVG (Shiba-Fukushima et al., 2014). pGEX4T-MIC60 (Akabane et al., 2016) was kindly provided by Drs. T. Oka (Rikkyo University). *PINK1*$^{-/-}$ HeLa cell line (Kane et al., 2014) and ΔOTC/HeLa-Tet On cell line (Jin and Youle, 2013) were kindly provided by R. Youle (NIH).

Drug discovery screening

The Takeda cpd library (~45,000 cpds) was plated onto 1536-well plates using an Echo 555 liquid handler (Labcyte). The reporter cell suspension (5 μl; 5 x 10^5 cells/ml) was dispensed onto 1536-well plates using a Multidrop Combi (Thermo Fisher Scientific). The plates were incubated for 3 h at 37˚C. DMSO and 1 μM val were included in every plate as negative and positive controls, respectively. Cell viability was monitored using CellTiter-Fluor Cell Viability Assay reagents (Promega) and NL activity was sequentially measured using the Nano-Glo Luciferase Assay System (Promega). Plate readings for the HTS were performed using EnVison (PerkinElmer). The chemiluminescence signal intensities (NL activity) was normalized using fluorescence (cell density) and the candidates were subjected to a dose-response test (at 0.3, 3, and 10 μM). Cpds showing over 30% reduction from the negative control (DMSO) in the Parkin-expressing cells were further tested using an adherent cell format with different doses (3 and 10 μM). The resultant 31 cpds were further subjected to ΔΨm testing using 10 nM MitoTracker Red CMXRos (Thermo Fisher Scientific). Two cpds with ≤ 10% ΔΨm changes at 3 μM were selected.

Antibodies and reagents

The following antibodies were used for western blotting: anti-phospho-Ub (1:1000 dilution; Millipore, ABS1513), anti-PINK1 (1:1,000; Novus, BC100-494; or 1:1,000; Cell Signaling Technology, clone D8G3), anti-Ub (1:1,000; Ub2, made in-house, (Shiba-Fukushima et al., 2014)), anti-human Parkin (1:1,000; Cell Signaling Technology, clone PRK8), anti-Mfn1 (1:1,000; Abnova, clone 3C9), anti-Tom20 (1:1,000; Santa Cruz Biotechnology, FL-145 or F-10), anti-Tim23 (1:2,000; BD, clone 32/Tim23), anti-LDH (1:500; Santa Cruz Biotechnology, H-160), anti-GFP (1:1,000; MBL, code No. 598), anti-MBP (1:1,000; New England BioLabs, E8030), anti-OPA1 (1:1,000; BD, 612606), anti-TBK1 pS172 (1:1,000; Cell Signaling Technology, clone D52C2), anti-TBK1 (1:1,000; Cell Signaling Technology, clone D1B4), anti-TH (1:1,000; Millipore, clone LNC1), anti-OTC (1:1000; Santa Cruz Biotechnology, clone E-9), anti-Actin (1:10,000; Millipore, MAB1501), and anti-Hsp60 (1:10,000; BD Biosciences, clone 24/Hsp60; 1:1000; Cell Signaling Technology, clone D307). The following antibodies were used for immunocytochemistry: anti-Tom20 (1:1,000; Santa Cruz Biotechnology, FL-145 or F-10), anti-PINK1 (1:100 prepared in Takeda), and anti-TH (1:1,000; Abcam, ab76442).

Val, antimycin, and oligomycin A were purchased from Sigma-Aldrich, Santa Cruz bio, and Cayman Chemical, respectively.

In vitro kinase assay

An *in vitro* kinase assay for PINK1 was performed using maltose-binding protein (MBP) fusion-*T. castaneum* PINK1 (TcPINK1) and bovine ubiquitin (Sigma-Aldrich). MBP-TcPINK1 (500 nM) was preincubated with T0466 in 40 μl of kinase reaction buffer (50 mM Tris-HCl, pH 7.5, 0.1 mM EGTA, 10 mM $MgCl_2$, 2 mM DTT, and 2 mM ATP) for 30 min at 22˚C and further incubated with 1 μM of ubiquitin for 90 min at 30°C. For PKA kinase assay, the PKA catalytic subunit (2 units, Sigma-Aldrich, P2645) was preincubated with T0466 for 30 min at 22˚C in 40 μl of kinase reaction buffer (50 mM Tris-HCl, pH 7.5, 0.1 mM EGTA, 10 mM $MgCl_2$, 2 mM DTT, and 10 μCi γ-^{32}P ATP) and further incubated with 1 μM GST-MIC60 for 90 min at 30°C.

Thermal shift assay

Recombinant Parkin (0.58 μM, Ubiquigent) in a reaction buffer containing 20 mM HEPES, pH 7.6, 10 mM $MgCl_2$, 2 mM DTT, 1 mM EGTA, SYPRO Orange (1:30 dilution, Thermo Fisher Scientific) with or without cpds was incubated at 22˚C for 20 min and transferred into a MicroAmp Optical 384 plate (10 μl/well). Changes in a thermal stability of Parkin due to the addition of cpds was assessed as shifts of fluorescent signals using ABI7900 (Applied Biosystems). Raw data at 25-99˚C (at a rate of 1˚C/min) were retrieved using Genedata Screener for TSA, and melt curves were generated by plotting the fluorescent signal or the first derivative of the fluorescent signal as a function of temperature.

Biochemical fractionation of cultured cells

HeLa/GFP-Parkin cells treated with drugs were suspended in mitochondrial isolation buffer (220 mM mannitol, 70 mM sucrose, 20 mM HEPES-KOH pH 7.4, 1 mM EDTA and protease inhibitor cocktail [Roche]), then homogenized by 30 passages using a 26-G needle on ice. Homogenates were centrifuged at 700 *g* for 10 min at 4˚C to obtain a post-nuclear supernatant (PNS). The PNS was further centrifuged at 12,000 *g* for 15 min. The supernatant served as a cytosolic fraction. The resultant pellets were washed several times with the mitochondrial isolation buffer and served as a mitochondrial fraction.

Human iPSCs

Normal iPSC (201B7) lines were differentiated into dopaminergic neurons by our reported protocol (Shiba-Fukushima et al., 2017). Briefly, dissociated neurospheres were allowed to adhere to poly-L-ornithine (Sigma-Aldrich) and laminin (GIBCO)-coated cultured plates or coverslips (Matsunami) and cultured in media hormone mix containing B27 (GIBCO), 10 ng/ml brain-derived neurotrophic factor (BDNF; R&D systems), 10 ng/ml glial cell-derived neurotrophic factor (GDNF; R&D systems), 200 mM ascorbic acid (Sigma-Aldrich), 1 mM dibutyryl-cAMP (Sigma-Aldrich), 1 ng/ml TGF-β (R&D systems) and 10 μM DAPT (Sigma-Aldrich) for 14 days to allow for differentiation into dopaminergic neurons. iCell skeletal myoblasts (SKM-301-020-001-PT) were provided by Cellular Dynamics and cultured, according to the manufacturer's instructions. Cellular and mitochondrial toxicities were estimated using the Mitochondrial ToxGlo Assay kit (Promega) and a multimode plate reader ($Mithras^2$ LB943, Berthold technologies).

Imaging of cell cultures and western blotting

HeLa and stable cell line imaging was performed using cultures plated on imaging chambers that had been precoated with poly-L-ornithine- and fibronectin (Corning Falcon Chambered Cell Culture Slides, 354104, Thermo Fisher Scientific). For imaging of neuron and skeletal myoblast cultures, cells were plated on poly-L-ornithine- and fibronectin-coated coverslips and stained with antibodies as described (Shiba-Fukushima et al., 2012). The cells were imaged using a laser-scanning microscope system (TCS-SP5, Leica or Zeiss LSM880 with Airyscan). For line profile analysis of human dopaminergic neuron culture, image stacks were acquired at 1 μm intervals using a 40x oil immersion objective with the pinhole diameter set to 1 airy unit using Zeiss LSM880 with Airyscan. Images were reconstructed using a series of stacked images using Zen software (Zeiss) and fluorescence intensity of cross-sections was measured using ImageJ (Fiji) plot-profile tool. Cells were lysed on ice with lysis buffer containing 0.2% NP-40, 50 mM Tris (pH 7.4), 150 mM NaCl and 10% glycerol supplemented

with protease inhibitor (Nacalai) and phosphatase inhibitor (Nacalai) cocktails. Western blotting using cell lysate was performed using ECL prime solution (GE Healthcare) (Shiba-Fukushima et al., 2017). Blot images were obtained using an Image Quant LAS 4000 mini (GE Healthcare).

Crawling assay

Flies expressing *PINK1 shRNA* in the muscles (*MHC-GAL4, UAS-PINK1 RNAi*) have been reported elsewhere (Yang et al., 2006). Flies expressing *LacZ shRNA* (a gift from Dr. S. Kawabata at Kyusyu University) were used as a control. First instar larvae were raised until the third instar larval stage on 1% agarose plates, where a yeast chunk (600 mg yeast powder kneaded with 1 ml distilled water) containing cpds was mounted. The same amounts of DMSO were used as a cpd solvent in each group. Third instar larvae treated with cpds were placed on the center of a 2% agarose plate (100 mm diameter) and their movement recorded every 10 sec during for 2 min. The movement trace and velocity in the last 60 sec were analyzed by ImageJ (Fiji).

Mitochondrial morphology in *Drosophila*

For Fig. 4, Fig. S5 and Videos, filets of third instar larvae expressing *MHC-GAL4* driven *mitoGFP* and *PINK1 shRNA* or *LacZ shRNA* were prepared in the HL-3 solution (70 mM NaCl, 5 mM KCl, 20 mM $MgCl_2$, 5 mM trehalose, 115 mM sucrose, 5 mM HEPES, and 10 mM $NaHCO_3$, pH 7.2) and fixed with 4% paraformaldehyde/phosphate buffered saline. Muscle tissues were counterstained with 25 ng/ml TRITC-labeled phalloidin (Sigma-Aldrich) and 1 µg/ml DAPI (Dojindo) at 4˚C overnight. For Fig. 5, brain tissues of third instar larvae expressing *TH-GAL4* driven *mitoGFP* and *PINK1 shRNA* or *LacZ shRNA* were isolated in the HL-3 solution and fixed with 4% paraformaldehyde/phosphate buffered saline. Brain tissues were counterstained with anti-TH (in-house, 1:250) (Yang et al., 2006). Image stacks were acquired at 0.35 µm intervals using a 63x oil immersion objective with the pinhole diameter set to 1 airy unit by SP5 (Leica). Images were reconstructed using a series of stacked images with ImageJ Z-projection tool. The volume of mitochondrial aggregates was measured by using IMARIS software (ver. 9.5.0, Bitplane).

Measurement of mitochondrial ATP and Ca^{2+} dynamics in *Drosophila*

Tissue ATP contents were analyzed using the CellTiter-Glo® Luminescent Cell Viability Assay (Promega). After drug treatment, 100 µl CellTiter-Glo® Reagent was added to *Drosophila* larval homogenates in white 96-well plates and samples were incubated according to the manufacturer's instruction. The effects of bacterial flora in the gut were negligible in this assay, so whole bodies were subjected to ATP measurement. ATP levels were estimated as luminescence intensity measured using a Mithras^2 LB943 plate reader. Luminescence intensities were standardized to the amounts of tissue soluble proteins measured using the Pierce BCA Protein Assay Kit (Thermo Fisher Scientific).

UAS-mitoGCaMP6 is a construct with the N-terminus of GCaMP6s harbors the mitochondrial targeting sequence of *Drosophila* Hsp60 (CG12101, 1-64 aa). Transgenic flies carrying *UAS-mitoGCaMP6* were generated in the w^{1118} background (BestGene). Filets of third instar larvae expressing *mitoGCaMP6* and *PINK1 shRNA* or *LacZ shRNA* under the control of the *MHC-GAL4* driver were prepared in the HL-3 solution, including 2 mM Ca^{2+}, and positioned on a silicone plate using insect pins. Brain tissues were removed and axons of the motor neurons projecting to the abdominal segment three muscles were sucked by a glass electrode. Changes in mitoGCaMP6 fluorescence intensity when electrically stimulated by 2.5 V (500 msec duration) at 20 sec-intervals were recorded using an Eclipse FN1 microscope (Nikon) equipped with an electrical stimulation setup containing a SEN-3401 (Nihon koden) and SS-104J (Nihon koden). The amount of fluorescence change ($\Delta F/F$) was analyzed using ImageJ.

Quantification of PINK1 transcripts in *Drosophila*

Third-instar larval body-wall muscles were dissected after drug treatment. Total RNA purified with TRIzol (Thermo Fisher Scientific) from three larval pools was subjected to reverse transcription and subsequent quantitative PCR using SuperScript IV VILO and SYBR GreenER (Thermo Fisher Scientific). Quantitative PCR was performed with QuantStudio 7 Flex (Applied Biosystems) using the following primers: dPINK1(ex1)-Fw, 5'-GCGCAGCTATTGTAAACGTGATATACAC;

dPINK1(ex2)-Rv, 5’-TGAGGATGTTGTCGATGAACAATTTGC; RP49 Fw, 5’-CCAAGGACTTCATCCGCCACC; RP49 Rv, 5’-GCGGGTGCGCTTGTTCGATCC.

Statistical analysis

Error bars in graphs represent mean ± the standard error of the mean unless otherwise indicated. The exact sample size of each experiment is provided in the relevant figure legends. Two-tailed student’s *t*-test or one-way analysis of variance (ANOVA) was used to determine significant differences between two or among multiple groups, respectively, unless otherwise indicated. If a significant result was determined using ANOVA ($p < 0.05$), the mean values of the control and the specific test group were analyzed using a Tukey-Kramer test. Dunnett’s test was used to detect significant differences between two specific groups or among multiple groups.

Supplemental References

Akabane, S., Uno, M., Tani, N., Shimazaki, S., Ebara, N., Kato, H., Kosako, H., and Oka, T. (2016). PKA Regulates PINK1 Stability and Parkin Recruitment to Damaged Mitochondria through Phosphorylation of MIC60. Molecular cell *62*, 371-384.

Jin, S.M., and Youle, R.J. (2013). The accumulation of misfolded proteins in the mitochondrial matrix is sensed by PINK1 to induce PARK2/Parkin-mediated mitophagy of polarized mitochondria. Autophagy *9*, 1750-1757.

Kane, L.A., Lazarou, M., Fogel, A.I., Li, Y., Yamano, K., Sarraf, S.A., Banerjee, S., and Youle, R.J. (2014). PINK1 phosphorylates ubiquitin to activate Parkin E3 ubiquitin ligase activity. The Journal of cell biology *205*, 143-153.

Shiba-Fukushima, K., Arano, T., Matsumoto, G., Inoshita, T., Yoshida, S., Ishihama, Y., Ryu, K.Y., Nukina, N., Hattori, N., and Imai, Y. (2014). Phosphorylation of mitochondrial polyubiquitin by PINK1 promotes Parkin mitochondrial tethering. PLoS genetics *10*, e1004861.

Shiba-Fukushima, K., Imai, Y., Yoshida, S., Ishihama, Y., Kanao, T., Sato, S., and Hattori, N. (2012). PINK1-mediated phosphorylation of the Parkin ubiquitin-like domain primes mitochondrial translocation of Parkin and regulates mitophagy. Scientific reports *2*, 1002.

Shiba-Fukushima, K., Ishikawa, K.I., Inoshita, T., Izawa, N., Takanashi, M., Sato, S., Onodera, O., Akamatsu, W., Okano, H., Imai, Y., *et al.* (2017). Evidence that phosphorylated ubiquitin signaling is involved in the etiology of Parkinson's disease. Human molecular genetics *26*, 3172-3185.

Yang, Y., Gehrke, S., Imai, Y., Huang, Z., Ouyang, Y., Wang, J.W., Yang, L., Beal, M.F., Vogel, H., and Lu, B. (2006). Mitochondrial pathology and muscle and dopaminergic neuron degeneration caused by inactivation of Drosophila Pink1 is rescued by Parkin. Proceedings of the National Academy of Sciences of the United States of America *103*, 10793-10798.
